# Importance of the CEP215-Pericentrin Interaction for Centrosome Maturation during Mitosis

**DOI:** 10.1371/journal.pone.0087016

**Published:** 2014-01-22

**Authors:** Seongjae Kim, Kunsoo Rhee

**Affiliations:** Department of Biological Sciences, Seoul National University, Seoul, Korea; Cancer Research UK London Research Institute, United Kingdom

## Abstract

At the onset of mitosis, the centrosome undergoes maturation, which is characterized by a drastic expansion of the pericentriolar material (PCM) and a robust increase in microtubule-organizing activity. CEP215 is one of the major PCM components which accumulates at the centrosome during mitosis. The depletion phenotypes indicate that CEP215 is essential for centrosome maturation and bipolar spindle formation. Here, we performed a series of knockdown-rescue experiments to link the protein-protein interaction properties of CEP215 to its biological functions. The results showed that CEP215 and pericentrin, another major PCM component, is interdependent for their accumulation at the spindle poles during mitosis. As a result, The CEP215-pericentrin interaction is required for centrosome maturation and subsequent bipolar spindle formation during mitosis. On the other hand, CEP215 interaction with γ-tubulin is dispensable for centrosome maturation. Our results provide an insight how PCM components are assembled to form a spindle pole during mitosis.

## Introduction

The centrosome in most animal cells functions as a major microtubule organizing center and controls cellular morphology, migration and subcellular transport. The centrosome consists of a pair of centrioles surrounded by pericentriolar material (PCM), in which the minus ends of microtubules are embedded. The amount of PCM and microtubule organizing activity of the centrosome fluctuate in parallel during the cell cycle. At the onset of mitosis, the centrosome matures with a robust increase in centrosomal γ-tubulin. The failure of this process causes defects in bipolar spindle formation and chromosome congression. Selected PCM components, including pericentrin [1], [2], CEP215/CDK5RAP2 [3], [4], NEDD1/GCP-WD [5], [6] and CEP192 [7], [8] are known to be critical for γ-tubulin recruitment to the centrosome. However, the means by which these PCM components accumulate and organize to form a mature centrosome is not fully understood.

Importance of CEP215 in mitotic spindle formation has been proposed since depletion of CEP215 reduced the γ-tubulin levels in the mitotic centrosomes and eventually caused defects in bipolar spindle formation [3], [4], [9]. Centrosomin, a *Drosophila* homologue of CEP215, also plays critical roles in proper spindle pole formation [10], [11]. Sufficient amounts of PCM components were not recruited to the mitotic centrosomes of the *centrosomin* loss-of-function mutant flies [11], [12]. Lucas and Raff [13] reported another *centrosomin* phenotype in which the centrioles were detached from the juxtaposed PCM layer; as a result, they travelled around and sometimes escaped from the spindle poles during mitosis. Detachment of the centrosomes from mitotic spindle poles was also reported in chicken DT40 cells with a CEP215-truncated mutant and in CEP215-depleted human culture cells [4], [14]. Mto1, a centrosomin-related protein in fission yeasts, is important for γ-tubulin recruitment to the functional microtubule organizing centers (MTOCs) [15], [16], [17]. Involvement of CEP215 in other centrosomal behaviors, such as centrosome cohesion and centriole engagement, was also reported [18], [19].

CEP215 and its homologues interact with selected members of PCM components for their functions [20]. Two conserved domains called Cnn motif 1 and 2 (CM1 and CM2) in CEP215 are binding sites for γ-tubulin and pericentrin, respectively [3], [21], [22], [23]. CM1 in CEP215, centrosomin and Mto1 are essential for γ-tubulin recruitment to the centrosome and MTOCs [3], [12], [17]. CM1 is also essential for attachment of the centrosomes to mitotic spindle poles as shown in the *CEP215* mutant chicken DT40 cells [14]. CM2 is located at C-terminal end of CEP215 and is known to interact with pericentrin and AKAP450 [22], [23]. However, it is unclear whether these interactions contribute to PCM accumulation during centrosome maturation or not.

Pericentrin is a large coiled-coil protein that serves as a scaffold for recruiting a number of PCM proteins [1], [9]. Pericentrin is required for centrosome maturation because its centrosomal level increases at the onset of mitosis, and its depletion results in a significant reduction of PCM components at spindle poles and leads to monopolar spindles [1], [2], [9]. Recently, super-resolution microscopic observations have suggested that pericentrin plays a pivotal role in the formation of the toroidal structure in interphase PCM and the expansion of the PCM lattice during mitosis [24], [25]. However, the means by which pericentrin functions as a scaffold and specifically interacts with PCM components for centrosome maturation remains obscure.

In this study, we investigated the molecular mechanisms of CEP215 function during centrosome maturation. Importantly, we attempted to link the protein-protein interaction properties of CEP215 with its biological functions. Our results indicate that CEP215 interaction with pericentrin is critical for efficient PCM accumulation during centrosome maturation.

## Results

### CEP215 Interaction with Pericentrin for its Localization and Spindle Pole Formation

**Figure 1:**
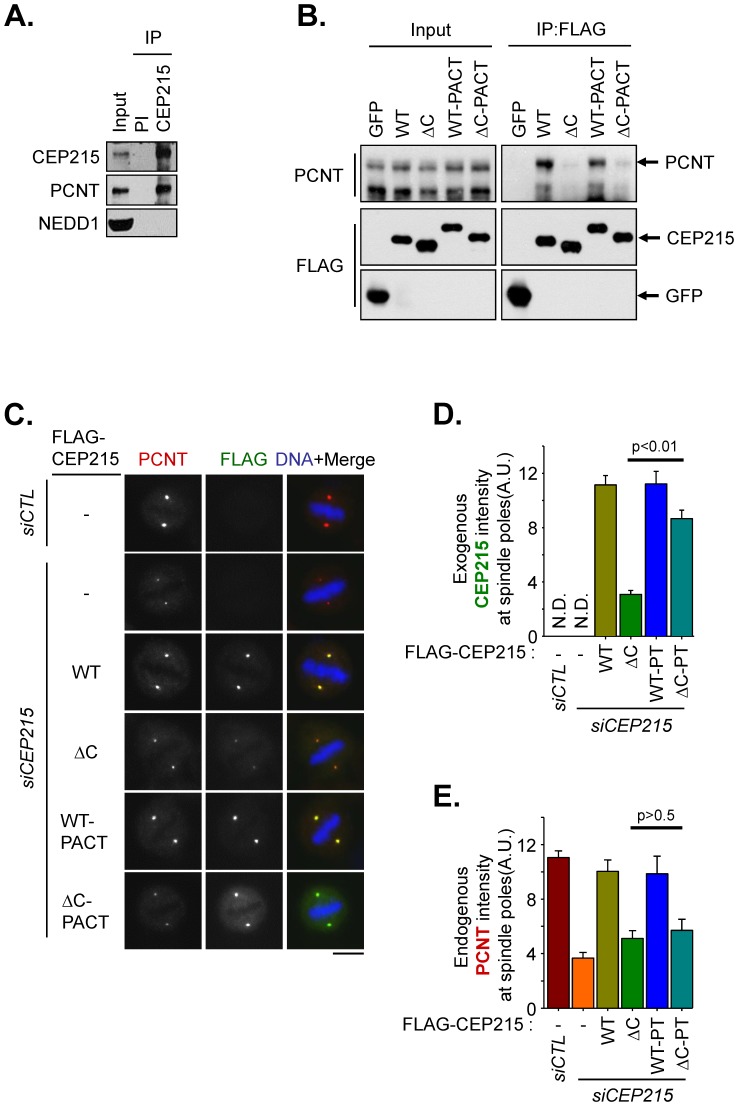
Rescue experiments with CEP215-truncated mutants devoid of the pericentrin-interaction domain. (A) Mitotic HEK293T cells were subjected to immunoprecipitation with pre-immune (PI) or CEP215-specific (CEP215) serum followed by immunoblot analysis with antibodies specific to CEP215, pericentrin and NEDD1. (B) HEK293T cells transfected with FLAG-tagged GFP, CEP215 (WT) and CEP215^Δ1726–1893^ (ΔC), CEP215-PACT (WT-PACT) and CEP215^Δ1726–1893^-PACT (ΔC-PACT) were treated with STLC for 16 h to synchronize the cells at mitosis. The mitotic cell lysates were subjected to immunoprecipitation with FLAG resin followed by immunoblot analysis with pericentrin and FLAG antibodies. (C) CEP215-depleted HeLa cells were rescued with FLAG-tagged CEP215 (WT) and CEP215^Δ1726–1893^ (ΔC), CEP215-PACT (WT-PACT) and CEP215^Δ1726–1893^-PACT (ΔC-PACT). The cells were treated with RO3306 for 16 h and subsequently removed for 40 min to allow accumulation of mitotic cells. (C) The cells were coimmunostained with pericentrin (red) and FLAG (green) antibodies. Scale bar, 10 µm. The intensities of ectopic CEP215 (D) and endogenous pericentrin (E) at the spindle poles were quantified in more than 40 cells per group in three independent experiments. Error bars, SEM. The paired t-test was performed with p values indicated.

In this work, we examined importance of CEP215-pericentrin interaction in mitotic cells. First, we confirmed physical interaction between the endogenous CEP215 and pericentrin proteins with a coimmunoprecipitation assay using mitotic cell lysates (Figure 1A). Then, we wished to make sure that the ectopic CEP215 proteins can interact with endogenous pericentrin in mitotic cells. The immunoblot and immunostaining analyses revealed that ectopic CEP215 proteins were expressed in comparable levels to the endogenous one (Figures S1A and S2). Furthermore, we observed that endogenous pericentrin was coimmunoprecipitated with the wild type FLAG-CEP215 but not with FLAG-CEP215^ΔC^ which lacks the CM2 domain (Figure 1B). Next, we performed knockdown-rescue experiments with the FLAG-CEP215 proteins. The endogenous CEP215 was efficiently depleted with specific siRNA transfection (Figures S1A and S3). Ectopic FLAG-CEP215 was prominently detected at the spindle poles of mitotic cells, whereas FLAG-CEP215^ΔC^ was not prominent at the spindle poles (Figures 1C, D and S2). The centrosomal level of endogenous pericentrin was reduced in CEP215-depleted cells and was recovered with ectopic FLAG-CEP215 (Figure 1C and E). However, the centrosomal pericentrin levels were not fully recovered with FLAG-CEP215^ΔC^ (Figure 1C and E). In order to enhance centrosomal localization of FLAG-CEP215^ΔC^, we fused the PACT domain to FLAG-CEP215^ΔC^ (FLAG-CEP215^ΔC^-PACT). Addition of the PACT domain does not affect the interaction property of FLAG-CEP215 proteins with pericentrin (Figure 1B). As expected, FLAG-CEP215^ΔC^-PACT was located at the centrosome in a comparable level to FLAG-CEP215 (Fig. 1C, D and S2). However, the centrosomal pericentrin levels were still not recovered with FLAG-CEP215^ΔC^-PACT (Fig. 1C and E). These results indicate that the CEP215-pericentrin interaction is critical for centrosomal localization of pericentrin in mitotic cells.

**Figure 2:**
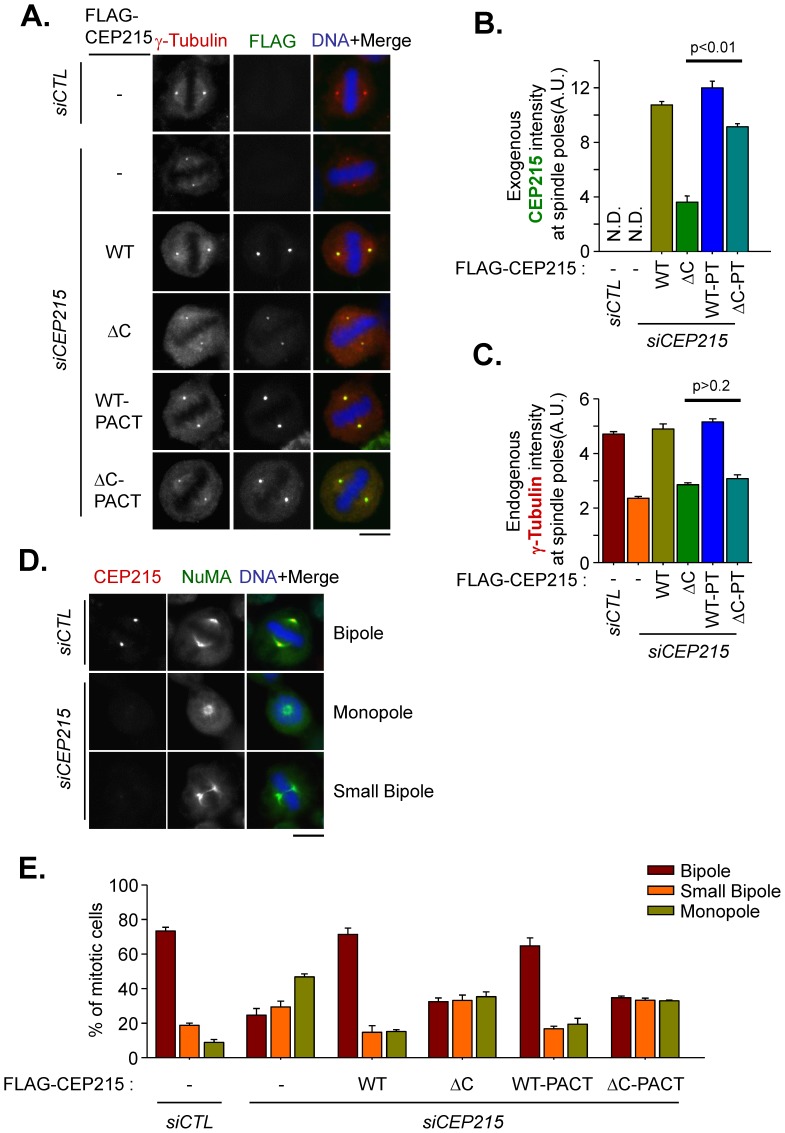
Spindle defects in cells rescued with the CEP215-truncated mutants. (A–C) CEP215-depleted HeLa cells were rescued with FLAG-tagged CEP215 (WT), CEP215^Δ1726–1893^ (ΔC), CEP215-PACT (WT-PACT) and CEP215^Δ1726–1893^-PACT (ΔC-PACT). The cells were treated with RO3306 for 16 h and subsequently removed for 40 min to allow accumulation of mitotic cells. The cells were coimmunostained with γ-tubulin (red) and FLAG (green) antibodies. Scale bar, 10 µm. The intensities of ectopic CEP215 (B) and endogenous γ-tubulin (C) at the spindle poles were quantified in more than 40 cells per group in three independent experiments. Error bars, SEM. The paired t-test was performed with p values indicated. (D) CEP215-depleted HeLa cells were coimmunostained with CEP215 (red) and NuMA (green) antibodies. The phenotype of the bipolar spindle was categorized as bipole, small bipole or monopole based on the NuMA staining patterns. Scale bar, 10 µm. (E) The CEP215-depleted HeLa cells were rescued with the FLAG-tagged CEP215 (WT), CEP215^Δ1726–1893^ (ΔC), CEP215-PACT (WT-PACT) and CEP215^Δ1726–1893^-PACT (ΔC-PACT). The cells were treated with RO3306 for 16 h and released with STLC for additional 1 h to arrest the cells at prometaphase. Then, the cells were washed and re-incubated with MG132 for 1.5 h to avoid the cells progress to anaphase. The cells were coimmunostained with FLAG and NuMA antibodies. The phenotype of the bipolar spindle was categorized as bipole, small bipole or monopole based on the NuMA staining patterns.

The centrosomal level of γ-tubulin reflects the centrosome maturation status. Therefore, we determined the centrosomal γ-tubulin levels in FLAG-CEP215-rescued mitotic cells. The results showed that the centrosomal γ-tubulin levels were significantly reduced in the CEP215-depleted cells and rebounded in the FLAG-CEP215-rescued cells (Figure 2A–C). On the other hand, neither FLAG-CEP215^ΔC^ nor FLAG-CEP215^ΔC^-PACT rescued the centrosomal γ-tubulin levels (Figure 2A–C). We also determined the mitotic spindle formation activity in the FLAG-CEP215-rescued cells. The morphology of mitotic spindles was categorized as bipole, small bipole or monopole based on the immunostaining pattern of NuMA (Figures 2D and S4). The results showed that FLAG-CEP215 and FLAG-CEP215-PACT successfully rescued the defects in bipolar spindle formation whereas FLAG-CEP215^ΔC^ and FLAG-CEP215^ΔC^-PACT did not (Figure 2E). Based on these results, we concluded that the physical interaction between CEP215 and pericentrin is critical for centrosome maturation and bipolar spindle formation during mitosis.

### Pericentrin also Needs to Interact with CEP215 for its Localization and Mitotic Spindle Pole Formation

**Figure 3:**
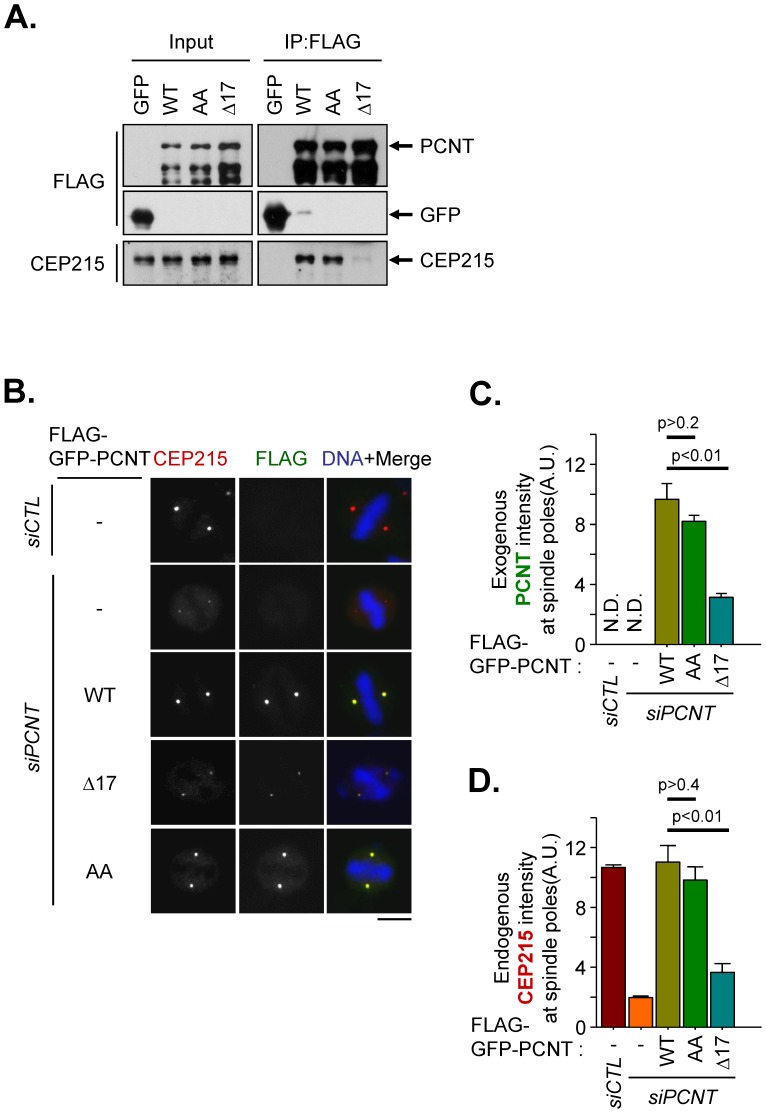
A pericentrin mutant devoid of CEP215 interaction. (A) HEK293T cells transfected with FLAG-tagged GFP, PCNT (WT), PCNT^1235,1241AA^ (AA) and PCNT^Δ2390–2406^ (Δ17) were treated with STLC for 16 h to synchronize the cells at mitosis. The mitotic cell lysates were subjected to immunoprecipitation with FLAG resin followed by immunoblot analysis with CEP215 and FLAG antibodies. (B–D) The pericentrin-depleted HeLa cells were rescued with FLAG-GFP-tagged PCNT (WT), PCNT^1235,1241AA^ (AA) and PCNT^Δ2390–2406^ (Δ17). The cells were treated with RO3306 for 16 h and subsequently removed for 40 min to allow accumulation of mitotic cells. The cells were coimmunostained with CEP215 (red) and FLAG (green) antibodies. Scale bar, 10 µm. The intensities of ectopic pericentrin (C) and endogenous CEP215 (D) at the spindle poles were quantified in more than 40 cells per group in three independent experiments. Error bars, SEM. The paired t-test was performed with p value indicated.

We used pericentrin mutants to examine functional significance of the CEP215-pericentrin interaction. We performed a series of coimmunoprecipitation assays with pericentrin-truncated mutants and eventually limited to 17 amino acids as a CEP215-interacting domain (2390–2406, PCNT^Δ17^). In fact, endogenous CEP215 was coimmunoprecipitated with ectopic FLAG-PCNT but hardly with FLAG-PCNT^Δ17^ (Figure 3A). The pericentrin-depleted HeLa cells were rescued with wild-type and mutant pericentrin proteins, and their subcellular localization was observed. Knockdown and ectopic expression of pericentrin were confirmed with immunoblot analyses (Figure S1B). The immunostaining results showed that FLAG-PCNT^Δ17^ was barely detectable at the spindle poles in comparison to FLAG-PCNT (Figure 3B and C). We then determined the intensity of CEP215 at the spindle poles in pericentrin-rescued cells. The results showed that the centrosomal levels of endogenous CEP215 were significantly reduced in pericentrin-depleted cells and were effectively rescued with ectopic FLAG-PCNT (Figure 3B and D). However, they were not rescued with FLAG-PCNT^Δ17^ (Figure 3B and D). These results confirmed that pericentrin and CEP215 must interact to each other for their localization to the mitotic spindle poles. We also utilized an alanine-substitution mutant of pericentrin at S1235 and S1241 (FLAG-PCNT^AA^), which cannot be phosphorylated by PLK1 during spindle pole formation [2]. A coimmunoprecipitation assay revealed that FLAG-PCNT^AA^ physically interacted with CEP215 in mitotic cells (Figure 3A). FLAG-PCNT^AA^ was localized at the spindle pole as much as FLAG-PCNT was (Figure 3B and C). At the same time, spindle pole levels of endogenous CEP215 in FLAG-PCNT^AA^-rescued cells were comparable to the control group (Figure 3B and D). These results indicate that the CEP215-pericentrin complex is formed independent of PLK1 phosphorylation of pericentrin.

**Figure 4:**
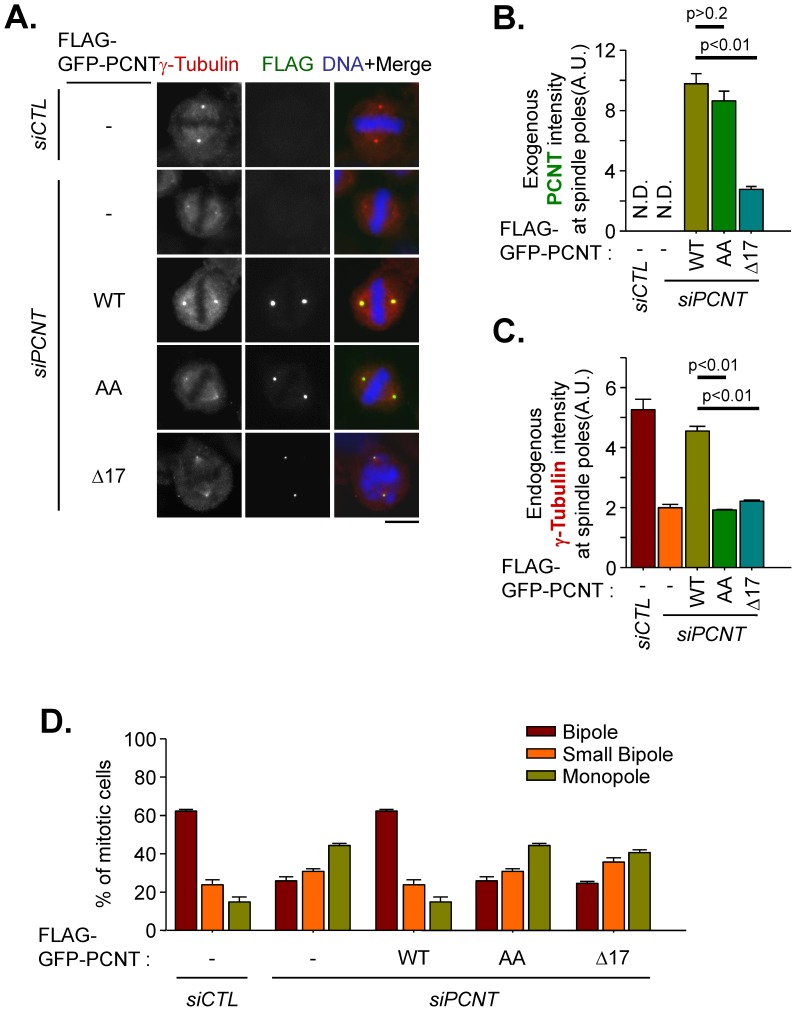
Spindle defects in cells rescued with the pericentrin mutant. (A–C) Pericentrin-depleted HeLa cells were rescued with FLAG-GFP-tagged PCNT (WT), PCNT^1235,1241AA^ (AA) and PCNT^Δ2390–2406^ (Δ17). The cells were treated with RO3306 for 16 h and subsequently removed for 40 min to allow accumulation of mitotic cells. The cells were coimmunostained with γ-tubulin (red) and FLAG (green) antibodies. Scale bar, 10 µm. The intensities of ectopic pericentrin (B) and endogenous γ-tubulin (C) at the spindle poles were quantified in more than 40 cells per group in three independent experiments. Error bars, SEM. The paired t-test was performed with p value indicated. (D) PCNT-depleted HeLa cells were rescued with FLAG-GFP-tagged PCNT (WT), PCNT^1235,1241AA^ (AA) and PCNT^Δ2390–2406^ (Δ17). The cells were treated with RO3306 for 16 h and released with STLC for additional 1 h to arrest the cells at prometaphase. Then, the cells were washed and re-incubated with MG132 for 1.5 h to avoid the cells progress to anaphase. The cells were coimmunostained with FLAG and NuMA antibodies. The phenotype of the bipolar spindle was categorized as bipole, small bipole or monopole based on the NuMA staining patterns.

To investigate the functional significance of the CEP215-pericentrin interaction for centrosome maturation, we determined the centrosomal γ-tubulin levels in FLAG-PCNT-rescued mitotic cells. The centrosomal γ-tubulin levels were significantly reduced in pericentrin-depleted cells and were effectively rescued with wild-type pericentrin (Figure 4A and C). However, the centrosomal γ-tubulin levels were not sufficiently rescued with either FLAG-PCNT^AA^ or FLAG-PCNT^Δ17^ (Figure 4A–C). We further determined the mitotic spindle formation activity in the pericentrin-rescued cells. Depletion of pericentrin resulted in abnormalities in bipolar spindle formation, including small bipole and monopole phenotypes (Figure S4). The wild-type pericentrin (FLAG-PCNT) successfully rescued the defects in spindle formation (Figures 4D); however, these phenotypes could not be rescued with either FLAG-PCNT^AA^ or FLAG-PCNT^Δ17^ (Figure 4D). These results reveal that pericentrin should interact with CEP215 and also should be phosphorylated by PLK1 to ensure centrosome maturation and subsequent bipolar spindle formation during mitosis.

### Physical Interaction of CEP215 with γ-tubulin is Dispensable for Spindle Pole Formation

**Figure 5:**
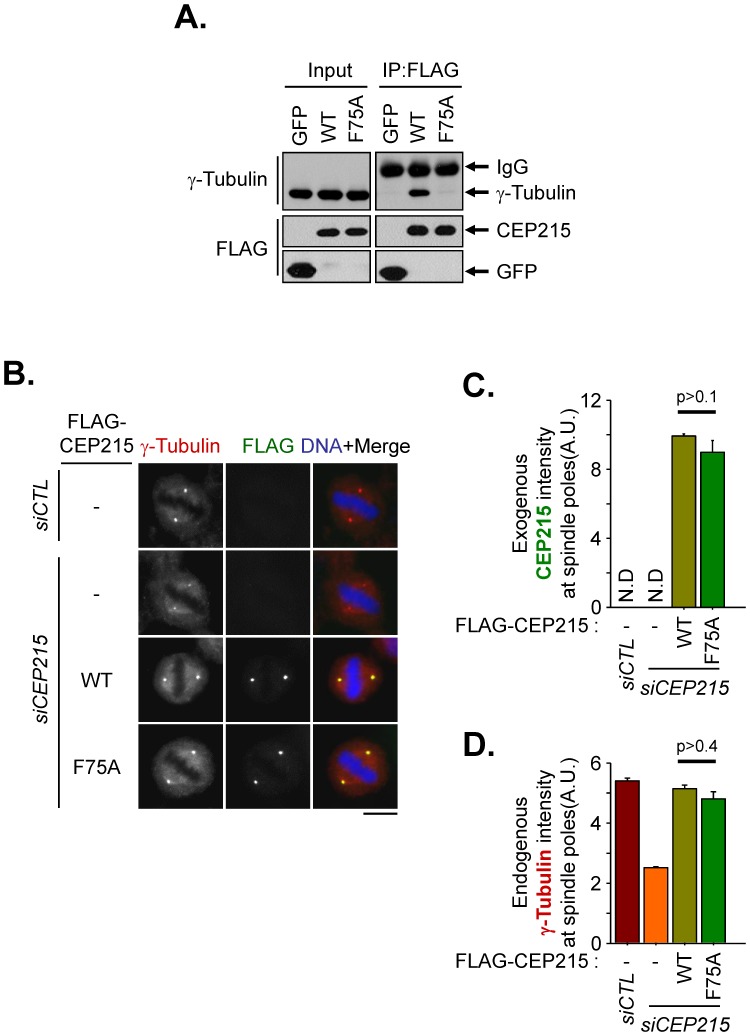
Spindle pole formation with cells rescued with a CEP215 mutant devoid of γ-tubulin interaction. (A) HEK293T cells transfected with FLAG-tagged GFP, CEP215 (WT) and CEP215^F75A^ (F75A) were treated with STLC for 16 h to synchronize the cells at mitosis. The mitotic cell lysates were subjected to immunoprecipitation with FLAG resin followed by immunoblot analysis with γ-tubulin and FLAG antibodies. (B) The CEP215-depleted cells were rescued with FLAG-tagged CEP215 (WT) and CEP215^F75A^ (F75A). The cells were treated with RO3306 for 16 h then removed for 40 min to allow accumulation of mitotic cells. The cells were coimmunostained with γ-tubulin (red) and FLAG (green) antibodies. Scale bar, 10 µm. (C, D) The intensities of ectopic CEP215 (C) and endogenous γ-tubulin (D) at the spindle poles were quantified in more than 40 cells per group in three independent experiments. Error bars, SEM. The paired t-test was performed with p value indicated.

CEP215 interacts with γ-tubulin through the CM1 domain and this interaction is known to contribute to centrosomal recruitment of γ-tubulin [3], [21]. In this work, we performed knockdown-rescue experiments to determine importance of CEP215-γ-tubulin interaction in mitotic cells. First, we confirmed that γ-tubulin was coimmunoprecipitated with ectopic CEP215 in mitotically arrested HEK293T cells (Figure 5A). Furthermore, we confirmed that endogenous γ-tubulin did not interact with an alanine substitution mutation of CEP215 at phenylalanine of the 75th residue (FLAG-CEP215^F75A^) (Figure 5A; [21]). We then performed rescue experiments to examine whether CEP215-γ-tubulin interaction contributes to γ-tubulin recruitment to the mitotic centrosome or not. CEP215-depleted HeLa cells were rescued with FLAG-CEP215 or FLAG-CEP215^F75A^ and coimmunostained with FLAG and γ-tubulin antibodies. The results showed that both FLAG-CEP215 and FLAG-CEP215^F75A^ were successfully localized at the spindle poles (Figure 5B and C). Concurrently, the centrosomal γ-tubulin levels in FLAG-CEP215^F75A^-rescued cells were comparable to those in FLAG-CEP215-rescued cells (Figure 5B and D). Taken together, we conclude that the physical interaction of CEP215 with γ-tubulin is dispensable for γ-tubulin recruitment to spindle poles during mitosis.

## Discussion

Physical interaction between CEP215 and pericentrin has been previously reported [22], [23]. However, importance of this interaction during mitosis has not been clearly elucidated yet. In this study, we revealed that the CEP215-pericentrin interaction is essential for their interdependent accumulation to mitotic centrosomes and eventually for spindle pole formation in mitotic cells.

The organizational features of PCM have been examined with sub-diffraction fluorescence microscopy. The analyses of interphase centrosome revealed that the C-terminal end of pericentrin is positioned at the centriole wall and radiates outward into the matrix [24], [25]. The PACT domain at the C-terminal end might play an important role in pericentrin attachment to the centriole wall in interphase cells [26]. Depletion of pericentrin results in reduction of CEP215 at the interphase centrosome [18], [22]. However, CEP215 depletion did not affect the centrosomal pericentrin levels in interphase cells [18], [22]. It was also reported that a CEP215 mutant without the pericentrin-interaction domain CM2 could not be located on the interphase centrosome [23]. These results suggest that the PCM structure in interphase centrosome is primarily maintained by pericentrin. CEP215 may be recruited to the centrosome through a specific interaction with pericentrin.

PCM accumulation mechanisms in mitotic cells are quite different from those in interphase cells. Pericentrin is still required for mitotic spindle pole formation as shown by the knockdown experiments [2], [9], [24]. Unlike interphase cells, CEP215 is also critical for spindle pole formation in mitotic cells. First, we and others showed that CEP215 depletion results in reduction of the centrosomal pericentrin levels in mitotic cells [9], [24]. Second, neither CEP215^ΔC^ nor PCNT^Δ17^was sufficiently accumulated to the mitotic centrosome because they were not able to form CEP215-pericentrin complexes. Finally, the centrosomal pericentrin levels remained low in CEP215^ΔC^-rescued mitotic cells. The centrosomal CEP215 levels also remained low in PCNT^Δ17^-rescued cells, indicating that the localization of CEP215 and pericentrin to spindle poles is interdependent during mitosis. Therefore, the CEP215-pericentrin complex is essential for centrosome maturation and eventually spindle pole formation during mitosis.

**Figure 6:**
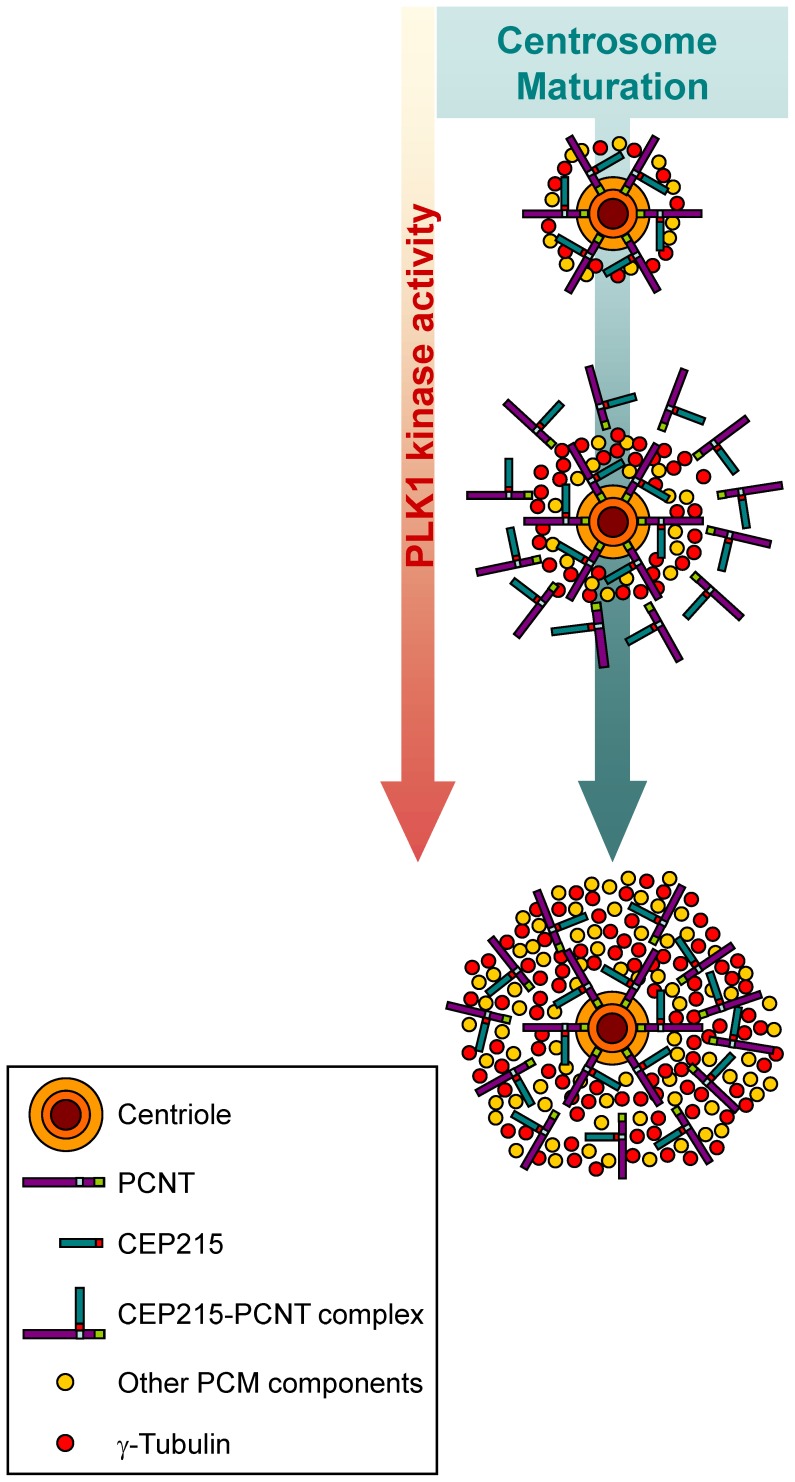
Model. At the onset of mitosis, the CEP215-pericentrin complex provides a proper environment for accumulation of the other PCM components in maturing centrosomes. Once pericentrin is phosphorylated by PLK1, additional PCM components are recruited to the centrosome, which becomes spindle poles with an eminent microtubule organizing activity.

We previously demonstrated that PLK1 phosphorylation of pericentrin is a critical step for PCM accumulation in centrosome maturation during mitosis [2]. CEP215 is already placed at the centrosome irrespective of the phosphorylation status of pericentrin [2]. Therefore, we propose a model in which the CEP215-pericentrin complex provides a proper environment for accumulation of the other PCM components in maturing centrosomes (Figure 6). Once pericentrin is phosphorylated by PLK1, additional PCM would be recruited to the centrosomes, which become spindle poles with an eminent microtubule organizing activity (Figure 6). If CEP215 was not properly positioned along with pericentrin, PCM components might not be recruited, and bipolar spindles might not be formed during mitosis [3], [4], [9]. The precise mechanisms by which the CEP215-pericentrin complex recruits PCM components to the spindle poles remain to be investigated. However, the physical interaction of CEP215 with γ-tubulin may not play a critical role in γ-tubulin recruitment to spindle poles because a CEP215 mutant that cannot interact with γ-tubulin (CEP215^F75A^) effectively rescued the CEP215-depletion phenotypes in mitotic cells. Instead, we propose that γTuRC might specifically interact with a group of PCM components that are recruited during centrosome maturation (Figure 6). In fact, additional PCM components such as NEDD1 are known to be essential for centrosomal recruitment of γTuRC for spindle pole formation [5], [6], [8], [9].

Mutations in *CEP215* are linked to primary microcephaly, a congenital disease that causes a substantial reduction in brain size at birth [27]. Natural *CEP215* mutant mice also have a small cerebral cortex that results from an overall reduction of neuronal layers [28]. The neuronal progenitor pool in CEP215 mutant mice may be prematurely depleted due to defects in centrosome maturation and/or mitotic spindle orientation [28]. Furthermore, there is evidence that CEP215 interaction with pericentrin is critical for the microcephaly phenotypes. First, CEP215 depletion leads to a premature neuronal differentiation, and pericentrin depletion phenocopies this effect [22]. Second, mutant CEP215 proteins in microcephaly patients have lost large regions of their C-termini, including the pericentrin-interacting domain [27], [29], [30]. In fact, mutations in pericentrin are linked to a number of congenital disorders, including microcephalic osteodysplastic primordial dwarfism type II [31], [32]. Thus, our research may provide insights into the molecular mechanisms of congenital brain disease.

## Materials and Methods

### Antibodies and Plasmids

The CEP215 and pericentrin antibodies have been previously described [4], [33]. Antibodies specific for NEDD1 (ab57336; Abcam), CEP192 (A302–324A; Bethyl Laboratories), γ-tubulin (sc-7396; Santa Cruz Biotechnology), FLAG (F3165; Sigma-Aldrich), FLAG (#2368; Cell Signaling), α-tubulin (ab18251; Abcam), NuMA (#NA09L; Calbiochem) and γ-tubulin (GTU-88; Sigma-Aldrich) were commercially purchased. Alexa Fluor 488-, 555- and 594-conjugated secondary antibodies (Invitrogen) were used for immunostaining. Anti-mouse IgG-HRP (A9044; Sigma-Aldrich) and anti-rabbit IgG-HRP (AP132P; Millipore) were used as secondary antibodies for immunoblotting.

The NCBI reference numbers of the human CEP215 and pericentrin cDNA sequences are NM_018249.5 and NM_006031.5, respectively. The CEP215^WT^ and CEP215^ΔC^ constructs were subcloned into the p3×FLAG-CMV10 vector. The PACT domain used in this study is the C-terminal region of the human pericentrin cDNA (9337–10011 bp). The wild-type and mutant pericentrin constructs (PCNT, PCNT^1235,1241AA^ and PCNT^Δ2390–2406^) were subcloned into the p3×FLAG-CMV10-GFP-myc vector. The coding sequences of CEP215 and pericentrin were silently mutated to become resistant to the corresponding siRNAs.

### Cell Culture and Transfection

HeLa cells and HEK293T cells were grown in DMEM supplemented with 10% FBS at 37°C. The siRNAs were transfected into HeLa cells using Lipofectamine RNAiMAX (Invitrogen) according to the manufacturer’s instructions. The plasmids were transfected into HeLa cells using FugeneHD (Promega). The siRNAs used in this study were *siCTL* (5′-GCA AUC GAA GCU CGG CUA CTT-3′), *siCEP215* (5′-GUG GAA GAU CUC CUA ACU AAA-3′) and *siPCNT* (5′-GCA GCU GAG CUG AAG GAG ATT-3′) [4], [34].

### Rescue Experiments and Cell Synchronization

For the rescue experiments, HeLa cells were initially transfected with siRNAs; 4 h later, the cells were transfected with the siRNA-resistant DNA constructs using FugeneHD. The cells were cultured for 24 h, treated with a CDK1 inhibitor, RO3306 (ALX-270-463; Enzo life sciences; 5 µM), for 16 h to arrest the cells at the G2-M boundary, and removed for 40 minutes. Most of the cells were at prometaphase to anaphase. To observe the bipolar spindle morphology, the cells were removed from RO3306 and subsequently treated with an Eg5 inhibitor, STLC (Tocris bioscience; 5 µM), for 1 h to arrest the cells at prometaphase. Then, the cells were washed three times with PBS and re-incubated in a medium containing 20 µM MG132 for 1.5 h to avoid progression to anaphase.

### Immunocytochemistry and Image Processing

HeLa cells were grown on 12-mm coverslips. The cells were fixed in methanol at −20°C for 10 min, washed with PBS, blocked in 3% bovine serum albumin (BSA) in PBS for 20 min, and incubated with the indicated primary antibodies for 1 h. The cells were washed with PBS with 0.3% TX-100 (PBST) and subsequently incubated with Alexa Fluor 488-, Alexa Fluor 555-, or Alexa Fluor 594-conjugated secondary antibodies (Invitrogen). The DNA was counterstained with DAPI. The samples were mounted in ProLong Gold antifade reagent (P36930; Invitrogen) and viewed on a fluorescence microscope (Olympus IX51) equipped with a CCD (Qicam fast 1394, Qimaging) camera. The images were analyzed using ImagePro 5.0 (Media Cybernetics, Inc.). The intensity of a specific protein was determined based on the sum of fluorescence intensity, and background intensity was subtracted. All statistical data were analyzed with SigmaPlot (Systat Software, Inc.).

### Immunoprecipitation and Immunoblotting Analyses

For coimmunoprecipitation experiments, HEK293T cells were transfected with DNA constructs using PEI. After 24 h, the cells were treated with STLC for 16 h and lysed on ice for 20 min with a lysis buffer (25 mM Tris-HCl [pH 7.4], 100 mM NaCl, 5 mM MgCl_2_, 5 mM NaF, 20 mM β-glycerophosphate and 0.5% NP-40) containing an appropriate amount of protease inhibitor cocktail (P8340; Sigma-Aldrich). The lysates were centrifuged at 12,000 rpm for 20 minutes at 4°C, and the supernatants were incubated with FLAG-M2 AffinityGel (A2220; Sigma-Aldrich) for 1.5 h at 4°C. The immunoprecipitates were then separated by SDS-PAGE, blotted onto a nitrocellulose membrane (Protran BA85; GE), and incubated with the indicated primary antibodies. HRP-conjugated secondary antibodies were used at a 1∶10,000 dilution. The immunoreactive bands were visualized using ECL solution.

## Supporting Information

Figure S1 **Immunoblot analyses to confirm the protein levels of knockdown and rescue groups of (A) CEP215 and (B) pericentrin.**
(TIF)

Figure S2 **Comparison of the level of ectopically rescued CEP215 with endogenous CEP215 at the mitotic spindle poles.** (A) CEP215-depleted HeLa cells were rescued with FLAG-tagged CEP215 (WT), F75A mutant CEP215 (F75A), CEP215^Δ1726–1893^ (ΔC), CEP215-PACT (WT-PACT) and CEP215^Δ1726–1893^-PACT (ΔC-PACT). The cells were treated with RO3306 for 16 h and subsequently removed for 40 min to allow accumulation of mitotic cells. The cells were coimmunostained with CEP215 (red) and FLAG (green) antibodies. Scale bar, 10 µm. (B) The intensities of CEP215 signal at the spindle poles were quantified in more than 20 cells per group in three independent experiments. Error bars, SEM. The paired t-test was performed with p value indicated.
(TIF)

Figure S3 **Reduction of PCM proteins in the spindle poles of CEP215-depleted mitotic cells.** (A) HeLa cells were transfected with nonspecific control (*siCTL*) or CEP215-specific (*siCEP215*) siRNAs for 48 h. The cell lysates were then harvested for immunoblot analysis with the indicated antibodies. (B) The CEP215-depleted HeLa cells were co-immunostained with γ-tubulin (red) and CEP215 (green) antibodies. Scale bar, 10 µm. (C) The CEP215-depleted mitotic cells were coimmunostained with CEP215 antibody (green) along with antibodies for γ-tubulin, pericentrin (PCNT), NEDD1 and CEP192 (red). Scale bar, 10 µm. (D) Relative intensities of γ-tubulin, pericentrin, NEDD1 and CEP192 at the spindle poles of CEP215-depleted cells were quantified in more than 40 cells per group in three independent experiments. Error bars, SEM.
(TIF)

Figure S4 **HeLa cells were transfected with** ***siCTL*** **,** ***siCEP215*** **and** ***siPCNT*** **. Forty-eight hours later, the cells were coimmunostained with α-tubulin (red) and NuMA (green) antibodies.** The phenotype of the bipolar spindle was categorized as bipole (completely separated NuMA), small bipole (arrowhead; inter-bridged NuMA) or monopole (round-shaped NuMA) based on the NuMA staining patterns. Scale bar, 10 µm.
(TIF)
