# Supplementary figures and images for: Importance of the CEP215-Pericentrin Interaction for Centrosome Maturation during Mitosis
Source: PLoS One. 2014 Jan 22;9(1):e87016. doi: 10.1371/journal.pone.0087016 (PMC3899370; doi:10.1371/journal.pone.0087016)

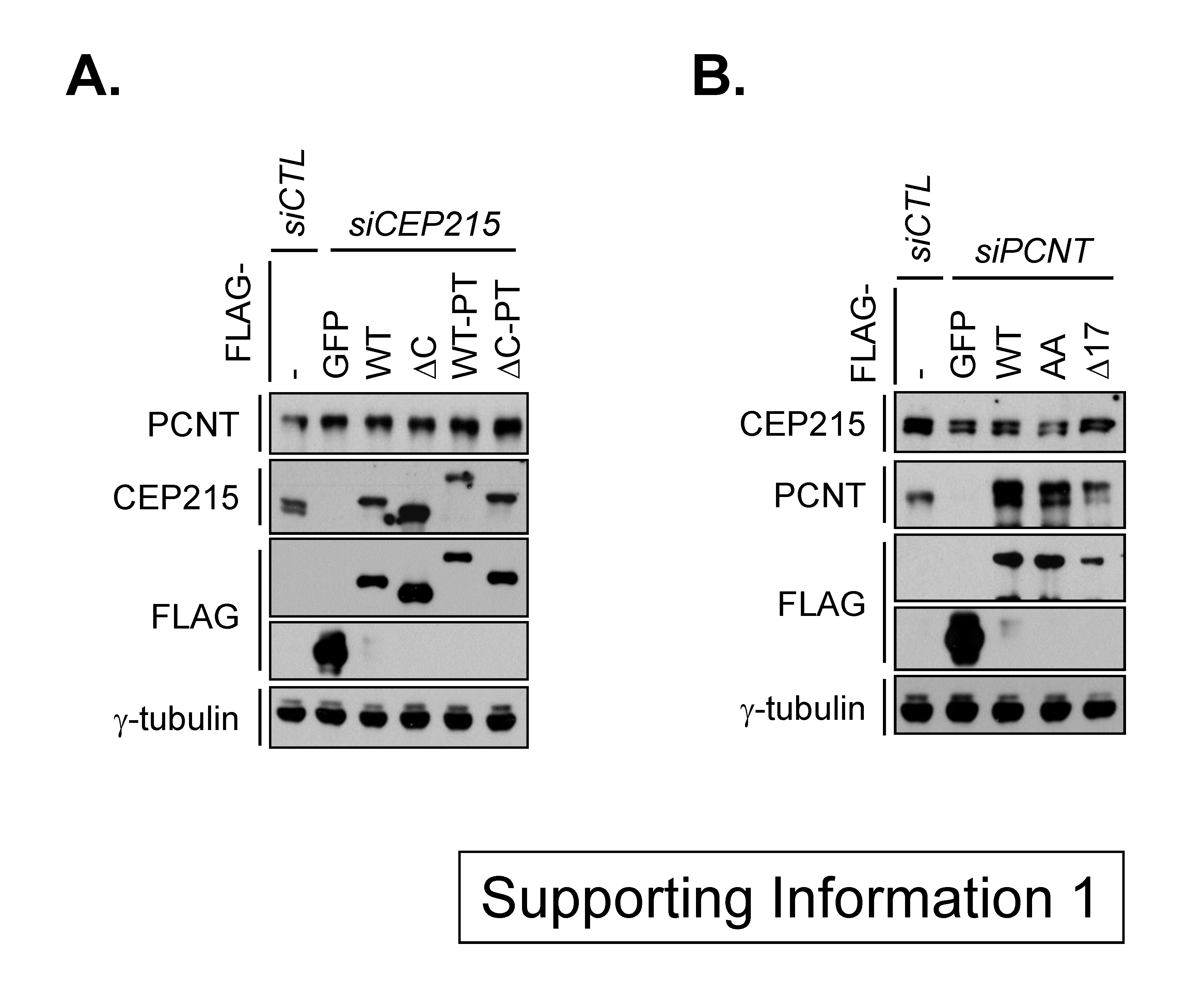

Supplement: Figure S1 — Immunoblot analyses to confirm the protein levels of knockdown and rescue groups of (A) CEP215 and (B) pericentrin. (TIF) [file pone.0087016.s001.tif]

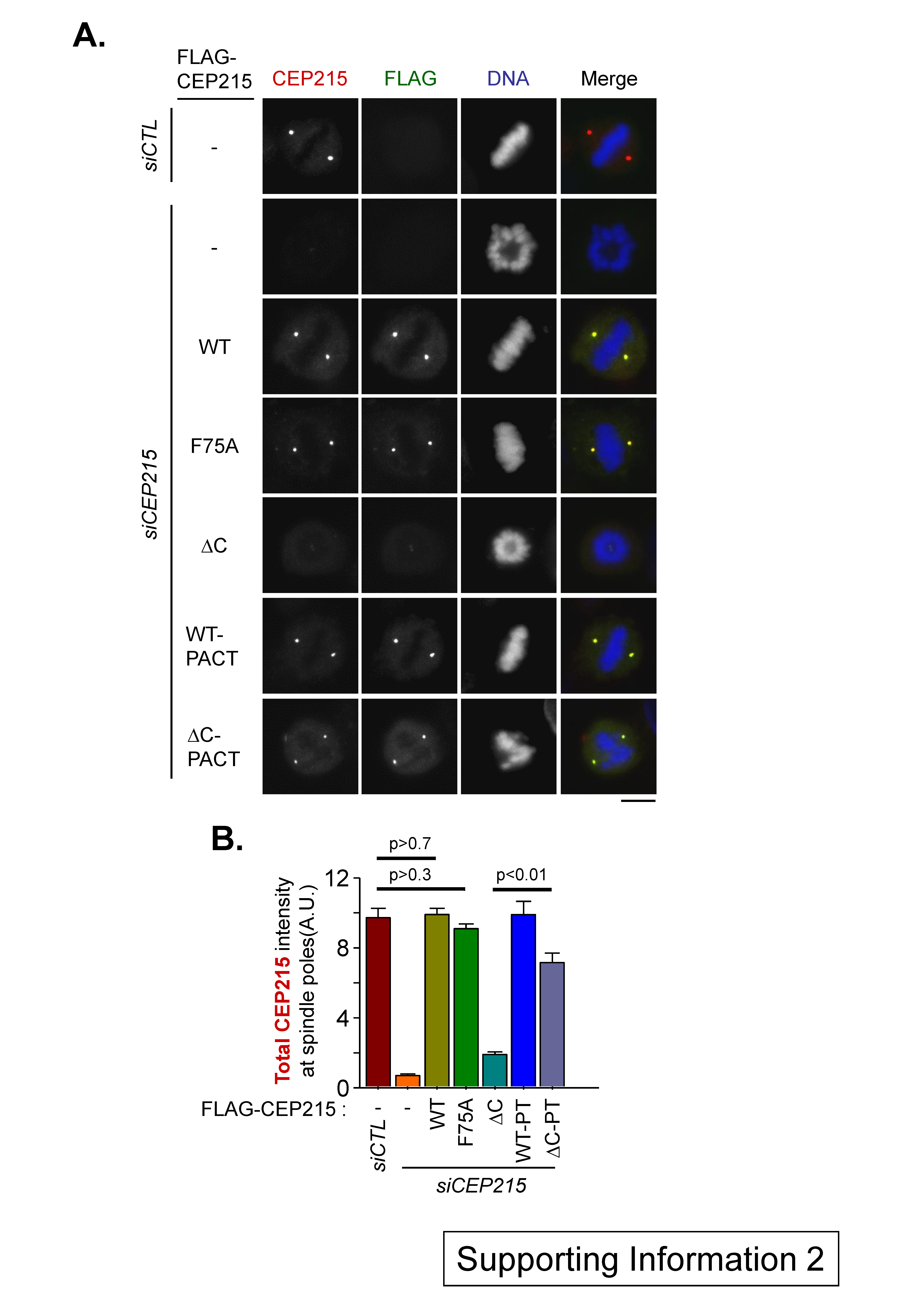

Supplement: Figure S2 — Comparison of the level of ectopically rescued CEP215 with endogenous CEP215 at the mitotic spindle poles. (A) CEP215-depleted HeLa cells were rescued with FLAG-tagged CEP215 (WT), F75A mutant CEP215 (F75A), CEP215Δ1726–1893 (ΔC), CEP215-PACT (WT-PACT) and CEP215Δ1726–1893-PACT (ΔC-PACT). The cells were treated with RO3306 for 16 h and subsequently removed for 40 min to allow accumulation of mitotic cells. The cells were coimmunostained with CEP215 (red) and FLAG (green) antibodies. Scale bar, 10 µm. (B) The intensities of CEP215 signal at the spindle poles were quantified in more than 20 cells per group in three independent experiments. Error bars, SEM. The paired t-test was performed with p value indicated. (TIF) [file pone.0087016.s002.tif]

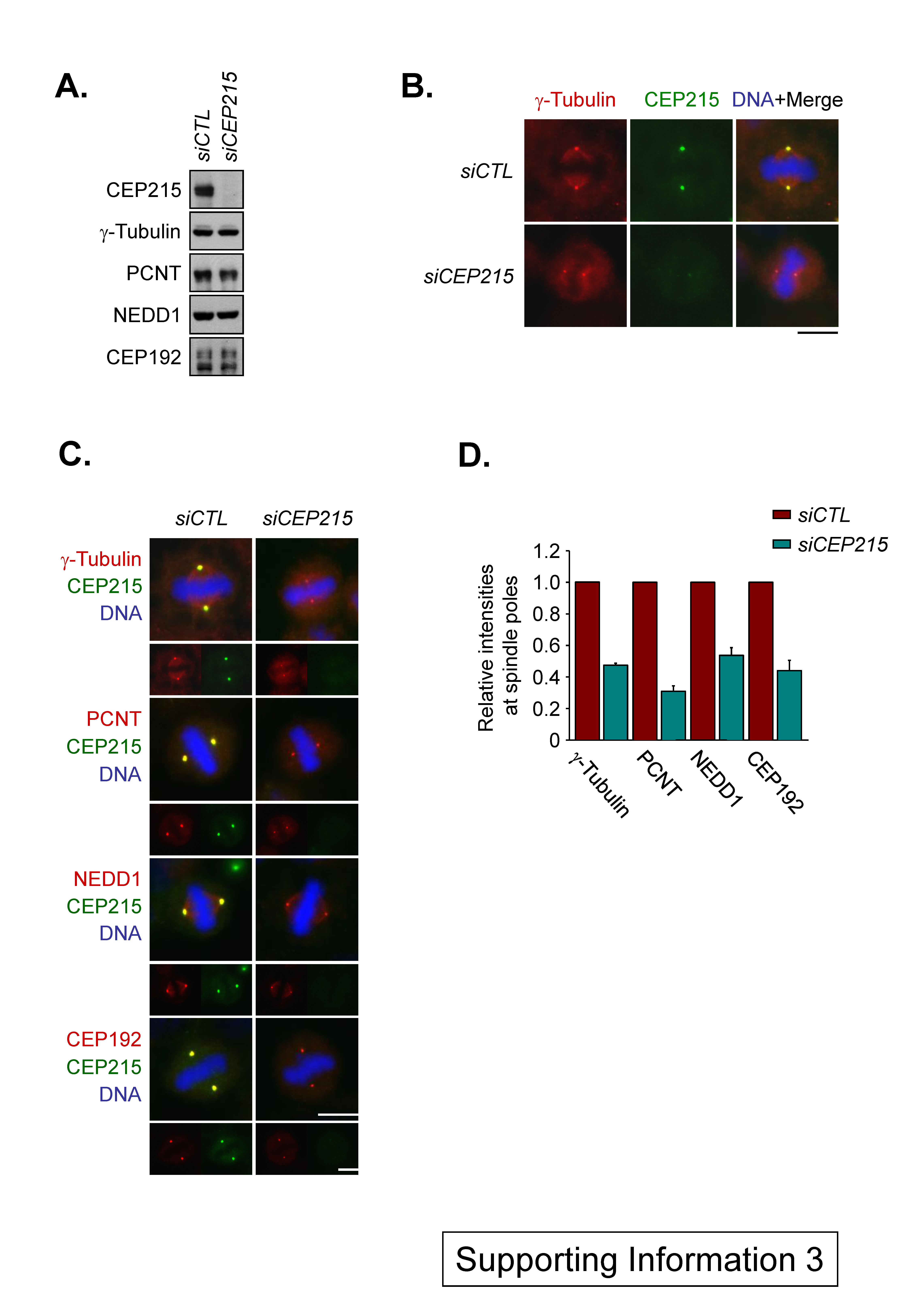

Supplement: Figure S3 — Reduction of PCM proteins in the spindle poles of CEP215-depleted mitotic cells. (A) HeLa cells were transfected with nonspecific control (siCTL) or CEP215-specific (siCEP215) siRNAs for 48 h. The cell lysates were then harvested for immunoblot analysis with the indicated antibodies. (B) The CEP215-depleted HeLa cells were co-immunostained with γ-tubulin (red) and CEP215 (green) antibodies. Scale bar, 10 µm. (C) The CEP215-depleted mitotic cells were coimmunostained with CEP215 antibody (green) along with antibodies for γ-tubulin, pericentrin (PCNT), NEDD1 and CEP192 (red). Scale bar, 10 µm. (D) Relative intensities of γ-tubulin, pericentrin, NEDD1 and CEP192 at the spindle poles of CEP215-depleted cells were quantified in more than 40 cells per group in three independent experiments. Error bars, SEM. (TIF) [file pone.0087016.s003.tif]

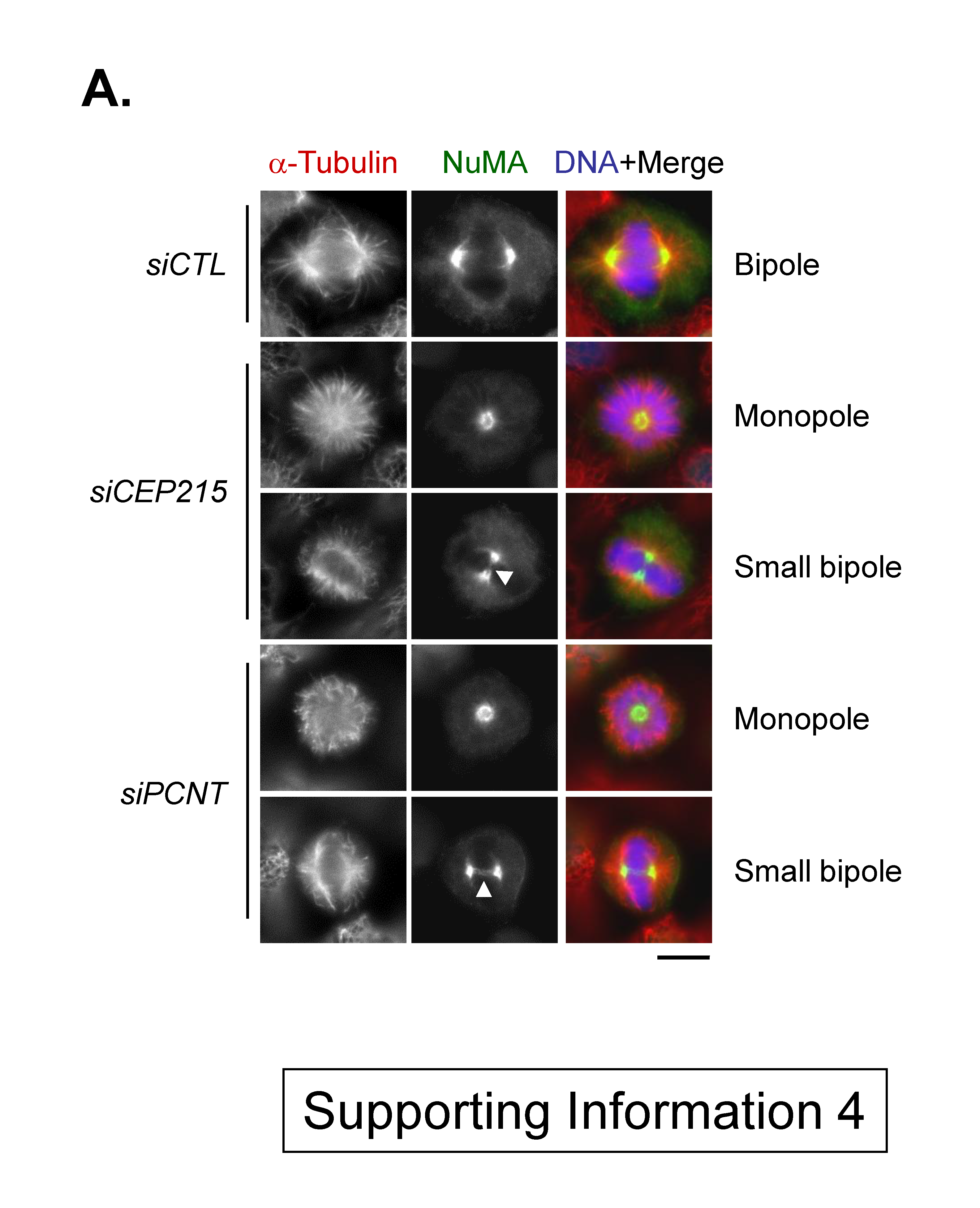

Supplement: Figure S4 — HeLa cells were transfected with siCTL , siCEP215 and siPCNT . Forty-eight hours later, the cells were coimmunostained with α-tubulin (red) and NuMA (green) antibodies. The phenotype of the bipolar spindle was categorized as bipole (completely separated NuMA), small bipole (arrowhead; inter-bridged NuMA) or monopole (round-shaped NuMA) based on the NuMA staining patterns. Scale bar, 10 µm. (TIF) [file pone.0087016.s004.tif]
